# Primary health care organization in the Covid-19 pandemic: scoping review

**DOI:** 10.11606/s1518-8787.2022056004374

**Published:** 2022-11-03

**Authors:** Breno Ribeiro Gonçalves da Silva, Ana Paula de Vechi Corrêa, Sílvia Carla da Silva André Uehara

**Affiliations:** I Universidade Federal de São Carlos Centro de Ciências Biológicas e da Saúde Departamento de Enfermagem São Carlos SP Brasil Universidade Federal de São Carlos. Centro de Ciências Biológicas e da Saúde. Departamento de Enfermagem. Graduação em Enfermagem. São Carlos, SP, Brasil; II Universidade Federal de São Carlos Centro de Ciências Biológicas e da Saúde Departamento de Enfermagem São Carlos SP Brasil Universidade Federal de São Carlos. Centro de Ciências Biológicas e da Saúde. Departamento de Enfermagem. Programa de Pós-Graduação em Enfermagem. São Carlos, SP, Brasil; III Universidade Federal de São Carlos Centro de Ciências Biológicas e da Saúde Departamento de Enfermagem São Carlos SP Brasil Universidade Federal de São Carlos. Centro de Ciências Biológicas e da Saúde. Departamento de Enfermagem. São Carlos, SP, Brasil

**Keywords:** Primary Health Care, organization & administration, COVID-19, prevention & control, Health Human Resource Training, Review

## Abstract

**OBJECTIVE:**

Mapping available scientific evidence on the organization of primary health care services and professionals during the Covid-19 pandemic.

**METHODS:**

This is a scoping review that followed the Joanna Briggs Institute method. Articles published in Portuguese, Spanish, and English from January 2020 to January 2021 in the CINAHL, Lilacs, Medline, PubMed, and Web of Science databases were included.

**RESULTS:**

We selected 24 articles that presented the reorganization of primary health care services and professionals to care suspected or confirmed Covid-19 cases. Coordination measures to tackle this disease in primary health care help to control its infection, especially by the active search for respiratory symptoms, the detection of new cases, and the monitoring of confirmed cases.

**CONCLUSION:**

This study presents an overview of how primary health care services and professionals organized themselves to tackle the Covid-19 pandemic, addressing adjustments in infrastructure and care flows, such as establishing specific Covid-19 care units, separating infected and non-infected patients, using telemedicine as an alternative modality of care, and monitoring cases by applications and phone.

## INTRODUCTION

In order to tackle the pandemic of the new coronavirus, health systems worldwide had to adjusted themselves to provide different responses against its infection, prevent its spread, and reduce the sequelae caused by Covid-19 in the population. Due to the high transmissibility rate of this disease, it rapidly spread worldwide, resulting, from the beginning of the pandemic to the first quarter of 2022, in more than 500 million confirmed cases and more than six million deaths worldwide^1^. During the same period, Brazil had more than 30 million confirmed cases and the number of deaths has already exceeded 660,000^2^.

The pandemic posed challenges for health systems, showing their weaknesses by exposing chronic funding and management problems^3^. Preparing these systems to tackle emerging infectious diseases, by evidence-based planning and strategies and coordination between all segments of a system and the government, is important.

In Brazil, primary health care (PHC) stands out since it is generally the main gateway for health services in the Brazilian Unified Health System (SUS). PHC is organized in order to solve most health problems of individuals and their families and played a fundamental role in the fight against the pandemic, as most infected people developed a mild form of the disease, which allowed their follow-up to be performed at this level of care^4^.

Considering actions to be developed to tackle Covid-19, especially in PHC, the knowledge of the territory, the access, the link between patients and health teams, the integrality of care, the monitoring of vulnerable families and suspected and mild cases of the disease allow the development of essential strategies to contain the pandemic and prevent the worsening of Covid-19^7^.

In Brazil, PHC remains a central point in the organization of the SUS, thus, strengthening and organizing it is necessary, in order to make it a pillar to tackle Covid-19, due to its capacity to link, manage, and monitor cases during the pandemic, and resume its routines after the pandemic, based on the context of the Country as a whole^7,8^.

Thus, PHC is essential to tackle Covid-19 worldwide, developing educational, preventive, health-promoting, care, and administrative actions. All health professionals and managers are responsible for organizing PHC in order to provide effective services and it must be done not only in health units, but throughout Brazil and at home.

Studies on the evolution and treatment of Covid-19, as well as its vaccines, have been published, however, mapping scientific evidence on studies on the organization of PHC health services and professionals and its effect on tackling the pandemic is necessary.

Thus, this study shows gaps on the topic and reflects on the organization of actions to mitigate Covid-19 at this level of care, allowing the planning of strategies to tackle this disease in order to provide a resolutive and quality care of the population. Therefore, this study aimed to map available scientific evidence on the organization of PHC services and professionals during the Covid-19 pandemic.

## METHODS

This is a scoping review that followed the Joanna Briggs Institute (JBI) method: (1) identification of the research question, (2) identification of relevant articles, (3) selection of articles, (4) data extraction, (5) separation, summarization, and reporting of results, and (6) dissemination of results^9^.

For the search, the JBI method, which consists of an acronym (PCC)—”P” for population, “C” for concept, and “C” for context—was used. This study considered “P” health professionals, “C” primary health care, and “C” organization and Covid-19, and it was developed based on the question: How was the organization of PHC services and health professionals during the Covid-19 pandemic?

Articles were searched in the following databases: US National Library of Medicine – National Institutes of Health (PubMed), Medical Literature Analysis and Retrieval System Online (Medline), Institute for Scientific Information (Web of Science), *Literatura Latino-Americana em Ciências da Saúde* (Lilacs – Latin American Literature in Health Sciences), and Cumulative Index to Nursing and Allied Health Literature (CINAHL). Articles were searched from March to May 2021 using keywords and their synonyms—organization, care, PHC, Covid-19, and health professionals—which were included in the *Descritor em Ciências da Saúde* (DeCS – Health Sciences Descriptors) and the Medical Subject Headings (MeSH) in different languages (Box 1).

**Box 1:** Search strategies used in the databases.

Database	Search strategies
PubMed	(((covid-19[Title]) OR coronavirus[Title])) AND (primary health care[Title] OR primary care[Title])) AND organization[Body - All Words]; (((covid-19[Title]) OR coronavirus[Title])) AND (primary health care[Title] OR primary care[Title])) AND assistance[Body - All Words]; (((covid-19[Title] OR coronavirus[Title])) AND (primary health care[Abstract] OR primary care[Abstract])) AND assistance[Body - All Words]; (((covid-19[Title] OR coronavirus[Title])) AND (primary health care[Abstract] OR primary care[Abstract])) AND organization[Body - All Words]; ((((covid-19[Title] OR coronavirus[Title])) AND (primary health care[Abstract] OR primary care[Abstract])) AND healthcare professionals[Abstract]) AND organization[Body - All Words]; (((covid-19[Title] OR coronavirus[Title])) AND (Family Health Strategy[Abstract] OR family health[Abstract])) AND organization[Body - All Words].
Medline	TI (covid-19 OR coronavirus OR coronavírus) AND AB (primary health care OR primary care OR atenção primária OR primeros auxilios) AND AB (organization OR organização OR organización); TI (covid-19 OR coronavirus OR coronavírus) AND TI (primary health care OR primary care OR atenção primária OR primeros auxilios) AND AB (organization OR organização OR organización); TI (covid-19 OR coronavirus OR coronavírus) AND AB (primary health care OR primary care OR atenção primária OR primeros auxilios) AND AB (assistance OR assistência OR asistencia); TI (covid-19 OR coronavirus OR coronavírus) AND AB (primary health care OR primary care OR atenção primária OR primeros auxilios) AND AB (organization OR organização OR organización) AND AB (healthcare professionals OR profesionales de la salud OR profissionais de saúde OR profissionais OR profesionales OR professionals); TI (covid-19 OR coronavirus OR coronavírus) AND AB (Family Health Strategy OR family health OR Estratégia Saúde da Família OR Saúde da Família OR salud familiar) AND AB (organization OR organização OR organización).
Web of Science	TÍTULO: (covid-19 OR coronavirus OR coronavírus) AND TÍTULO: (primary health care OR primary care OR atenção primária OR primeros auxilios) AND TÓPICO: (organization OR organização OR organización); TÍTULO: (covid-19 OR coronavirus OR coronavírus) AND TÓPICO: (primary health care OR primary care OR atenção primária OR primeros auxilios) AND TÓPICO: (organization OR organização OR organización); TÍTULO: (covid-19 OR coronavirus OR coronavírus) AND TÓPICO: (primary health care OR primary care OR atenção primária OR primeros auxilios) AND TÓPICO: (assistance OR assistência OR asistencia); TÍTULO: (covid-19 OR coronavirus OR coronavírus) AND TÓPICO: (primary health care OR primary care OR atenção primária OR primeros auxilios) AND TÓPICO: (organization OR organização OR organización) AND TÓPICO: (healthcare professionals OR profesionales de la salud OR profissionais de saúde OR profissionais OR profesionales OR professionals); TÍTULO: (covid-19 OR coronavirus OR coronavírus) AND TÓPICO: (Family Health Strategy OR family health OR Estratégia Saúde da Família OR Saúde da Família OR salud familiar) AND TÓPICO: (organization OR organização OR organización).
Lilacs	covid-19 OR coronavirus OR coronavírus [Title words] and primary health care OR primary care OR atenção primária OR primeros auxilios [Words] and organization OR organização OR organización [Words]; covid-19 OR coronavirus OR coronavírus [Palavras do título] and primary health care OR primary care OR atenção primária OR primeros auxilios [Palavras] and assistance OR assistência OR asistencia [Palavras]; covid-19 OR coronavirus OR coronavírus [Palavras do título] and primary health care OR primary care OR atenção primária OR primeros auxilios [Palavras] and healthcare professionals OR profesionales de la salud OR profissionais de saúde OR profissionais OR profesionales OR professionals [Palavras].
CINAHL	TI (covid-19 OR coronavirus OR coronavírus) AND AB (primary health care OR primary care OR atenção primária OR primeros auxilios) AND AB (organization OR organização OR organización); TI (covid-19 OR coronavirus OR coronavírus) AND AB (primary health care OR primary care OR atenção primária OR primeros auxilios) AND AB (organization OR organização OR organización) AND AB (healthcare professionals OR profesionales de la salud OR profissionais de saúde OR profissionais OR profesionales OR professionals); TI ( covid-19 OR coronavirus OR coronavírus ) AND AB ( Family Health Strategy OR family health OR Estratégia Saúde da Família OR Saúde da Família OR salud familiar) AND AB ( organization OR organização OR organización ); TI ( covid-19 OR coronavirus OR coronavírus ) AND AB ( primary health care OR primary care OR atenção primária OR primeros auxilios ) AND AB ( organization OR organização OR organización ) AND AB ( nursing OR nurse OR nursing care OR nursing practice OR enfermagem OR enfermería ).

Primary articles in Portuguese, English, and Spanish, published from January 1, 2020, to January 31, 2021, and indexed in the aforementioned databases were included. Protocols, editorials, systematic reviews, and articles with titles and abstracts that did not answer the research question, as well as information from websites and reported by the media, were excluded.

Later, references were exported to the StArt (State of the Art through Systematic Review*)* application for the selection of articles on two levels. The first selection was performed by the reading of titles and abstracts, followed by the reading of the full text. StArt was developed by the *Laboratório de Pesquisa em Engenharia de Software* (LaPES – Software Engineering Research Laboratory) of the Universidade Federal de São Carlos (UFSCar)^10^.

Eligible articles were fully recovered and evaluated by two researchers. In both phases, divergences were discussed until reaching a consensus and making the final selection—in this last moment, a third researcher participated. This study was based on Preferred Reporting Items for Systematic Reviews and Meta-analyses Extension for Scoping Reviews (PRISMA-ScR)^11^.

Authorship, journal name, country where the study was performed, country of publication, study design, main results, and gaps were the relevant information collected from each selected article. Results on bibliometric aspects that answer the research question are presented in boxes and in the main text of this scoping review.

## RESULTS

We found 1,262 articles in the databases used, however, we excluded 374 because they were duplicates, 833 after reading their titles and abstracts, and 31 after reading the full text. Thus, we studied 24 articles addressing the organization of PHC services and health professionals during the Covid-19 pandemic (Figure).

**Figure:**
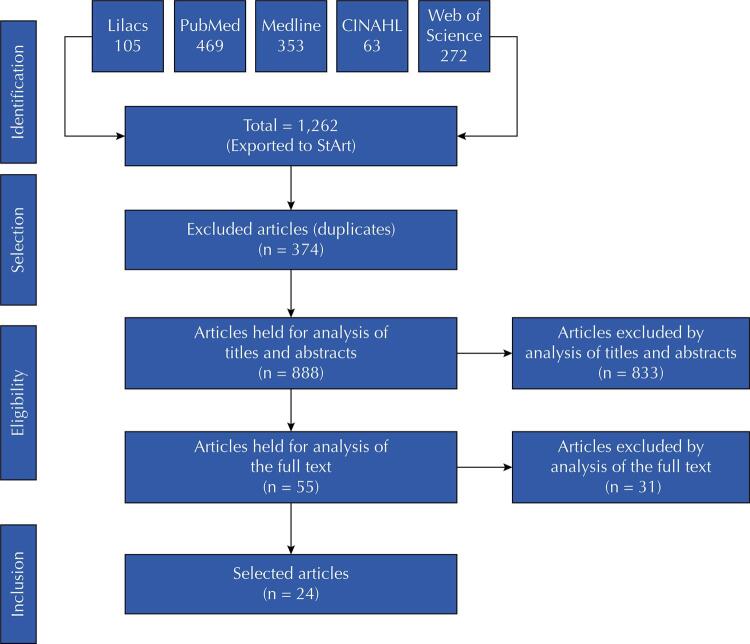
Reference flowchart: inclusion and exclusion of articles.

All articles included in this study were published from January 1, 2020, to January 31, 2021. Five of them (21%) were performed in Brazil, four (17%) in the United States, two (8%) in Oman, the United Kingdom, Italy, and Belgium, and one (4%) in Spain, India, Flanders, Saudi Arabia, Botswana, France, and Iceland. Regarding languages, 20 (83%) articles were published in English and four (17%) in Portuguese.

Among these 24 articles, nine were experience reports, five were observational studies, four were descriptive studies, two were cross-sectional studies, two were qualitative studies, and two were case studies (Box 2).

**Box 2:** Selected articles, according to place and year of publication, objective, type of study, and main results.

Author, year, and place	Objective	Type of study	Main results
Krist AH et al.^18^, 2020, United States	Presenting actions to be adopted by PHC during the pandemic, according to the protocol of the Centers for Disease Control and Prevention (CDC).	Descriptive study	The plan to tackle the Covid-19 pandemic in PHC was organized into six phases: 1) surveillance, notification, and monitoring of cases; 2) physical distancing, increasing virtual appointments, and postponing non-urgent appointments; 3) implementation of actions to flatten the epidemiological curve; 4) referral of only severe cases to hospitals; 5) care actions for convalescent patients; 6) addressing the consequences of the pandemic.
Morreel S et al.^19^, 2020, Belgium	Assessing the organization and characteristics of PHC appointments performed outside office hours and comparing them with those in the same period in 2019.	Observational study	By telephone screening, patients were classified as suspected or regular. All suspected Covid-19 cases were treated virtually, and, if necessary, referred to exclusive care units for Covid-19 suspected patients (Corona Units), or in home visits, or referred to emergency departments. In comparison with 2019, the workload increased due to phone calls, however, the number of face-to-face appointments decreased by 45%.
Fernandes LMM et al.^20^, 2020, Brazil	Analyzing the adaptation of a PHC center in Recife, which improved its telehealth and remote monitoring.	Case report	Reorganization of the internal flow of the unit, by separating symptomatic from asymptomatic patients; discontinuation of collective activities; maintenance of the follow-up of patients with chronic diseases; telemedicine and remote monitoring; and active surveillance actions promoted by the health team in the territory.
Sigurdsson EL et al.^21^, 2020, Iceland	Analyzing how PHC in Iceland changed its strategy to tackle the Covid-19 pandemic.	Observational descriptive study	Early detection of suspected cases; effective screening; separation of symptomatic and asymptomatic patients; maintenance of activities aimed at maternity and childcare; change from face-to-face care to telemedicine; alternative office hours. Change in the 10 main diagnoses—immunization, depression, hypothyroidism, and low back pain were no longer among the 10 main diagnoses. These changes showed a very solid PHC, with great flexibility in its organization.
Dias EG^22^, 2020, Brazil	Discussing the management of care and health education in PHC to tackle the Covid-19 pandemic.	Experience report	Immediate identification of respiratory symptoms; space reserved to wait for an appointment; discontinuation of some care activities; telemedicine and telemonitoring; home visit or face-to-face care, if necessary; health education by radio, sound cars, flyers, posters, social networks, and phone.
Vieira DS et al.^23^, 2020, Brazil	Developing an experience of organizational planning in nursing along with the family health team in a rural area of Igreja Nova, AL, during the Covid-19 pandemic.	Experience report	This study followed three strategies: •Community guidance on the problem of health prevention and promotion actions. •Permanent health education for the health team, in order to qualify professionals. •Organization of the provision of health services, establishment of an exclusive room to treat respiratory symptoms, and organization of the care of patients of programs in the unit.
Jacobson, NA et al.^28^, 2020, Brazil	Addressing a collaborative effort to care for a community during the Covid-19 pandemic from the development of a clinic.	Experience report	The authors analyzed the adaptation of a clinic with PHC exclusively for Covid-19 patients. Nurses determined if patients would need emergency care or care at the Covid-19 clinic by a virtual evaluation, by Zoom. Later, patients had a face-to-face appointment scheduled at the specific unit. In order to maximize social distancing, the physical space was adjusted.
Saint-Lary O et al.^29^, 2020, France	Analyzing how general physicians adapted their practices to ensure and maintain access to care during the epidemic phase.	Descriptive observational study	A total of 5,424 French general physicians were interviewed. Of them, 70.9% changed face-to-face care to remote care, 66.5% increased the number of remote appointments, and 42.7% created a specific flow for suspected Covid-19 patients. Of the 70.9% who adapted their practice, 91.7% used phones, 27.6% email, and 30.7% increased their use of video calls.
Majeed A et al.^30^, 2020, England	Assessing the response of primary health care for Covid-19 in the National Health Service (NHS) in England.	Descriptive observational study	Change from face-to-face care to remote care; care of suspected or confirmed Covid-19 patients in specific clinics; implementation of home visits specific for Covid-19 care; complete computerization of all National Health Service units; use of electronic medical records; online access of patients to health services (such as making appointments, asking for prescriptions, and seeing medical records); electronic sending of prescriptions, directly to the pharmacies of the patient’s choice.
Al Ghafri T et al.^32^, 2020, Oman	Assessing experiences and perceptions of PHC health professionals during the Covid-19 pandemic in relation to medical response experiences, sociocultural and religious reforms, psychological impressions, and lessons learned.	Qualitative and phenomenological study	Rapid reorganization of PHC services; use of technology; challenges of working in the Covid-19 pandemic; changes in social and religious standards; emergence of gaps in the access of vulnerable groups to health care; emergence of psychological disorders due to social distancing; management of corpses; exhaustion of health professionals; risk of exposure; and development of epidemiological and public health capacities, improving access to health care.
Blazey-Martin D et al.^33^, 2020, United States	Developing an innovative population management approach to remotely manage Covid-19 patients.	Experience report	An algorithm was used to guide screening decisions, including the frequency of contact with patients, depending on the day of the onset of symptoms and risk factors, and identify clinical findings that showed if patients were safe to stay at home or required further evaluation. These interventions, when performed on time, reduced the demand of hospitals and emergency care.
Verhoeven V et al.^34^, 2020, Flandres	Assessing the consequences of the Covid-19 outbreak experienced by physicians on the front line on the core competencies of family and community medicine.	Experience report	Screening and appointments performed by phone for all patients; clinical decision-making focused on respiratory evaluation and screening; impairment of acute care due to the change of focus to only Covid-19 and patients that did not seek care for problems that were not related to Covid-19; postponement of chronic care.
Karim SI et al.^35^, 2020, Saudi Arabia	Analyzing the strategies adopted to reorganize PHC during the Covid-19 pandemic in a hospital in the capital of Saudi Arabia.	Experience report	Services were adapted based on surveillance and case detection, clinical management, prevention of the spread, and maintenance of essential services. These changes were made by the Department of Family Medicine and all appointments started to be performed remotely, by WhatsApp and virtual clinics. Moreover, new means of communication, such as websites, portal messages, and social networking, started to be used.
Motlhatlhedi K et al.^36^, 2020, Botswana	Assessing the action of family health physicians during the pandemic.	Descriptive observational study	Family health physicians were responsible for infection control, disease prevention measures, identification of cases, dissemination of information, and continuity of medical care, especially for patients. The use of WhatsApp has expanded to include online webinars, as well as to disseminate information about Covid-19 from various sources and the national coordination center.
Sinha S et al.^37^, 2020, United States	Evaluating the implementation of a video care program at a large academic PHC clinic in New York.	Case study	In total, 1,030 video appointments were performed for 817 patients. Of them, 42% were due to Covid-19 symptoms and 58% due to acute or chronic conditions. Most patients were young adults, women, and people with commercial insurance. The degree of satisfaction was high (mean of 4.6 on a 5-point scale [SD = 0.97]).
Joy M et al.^38^, 2020, England	Assessing the responsiveness and prioritization of the type of PHC appointment for older adults during the Covid-19 pandemic.	Cross-sectional study	The rate of phone and video appointments more than doubled during the study period (106.0% and 102.8%, respectively). Face-to-face appointments decreased by 64.6% and home visits by 62.6%. This process coincided with national policy changes. The relative increase in the number of appointments was associated with people taking ≥ 10 medicines in comparison with those who did not take any.
Al Ghafri T et al.^39^, 2020, Oman	Assessing responses to PHC units from January to April 2020, including public health measures in Muscate, Oman.	Descriptive study	The number of appointments decreased from 115,324 in January to 109,719 in March 2020. Essential services were guaranteed in all health centers, especially for vulnerable groups, women, and children. Health centers were opened for 24 hours to ensure the force of testing and isolation. Telemedicine and virtual communications were adopted. Campaigns on the importance of social distancing and hand hygiene were promoted.
Bressy S^40^, 2020, Italy	Assessing the experience of PHC management to tackle Covid-19 in Italy during the beginning of the pandemic.	Experience report	Technological support and remote approach are essential to assess Covid-19 primary care. The use of telemedicine and the aid of technology allowed an efficient monitoring of patients at home, reducing inadequate hospitalizations, as patients were referred to a hospital only when necessary.
Mantovani W et al.^41^, 2020, Italy	Assessing the organization and the role of the Department of Prevention of the Local Health Unit of Trento in the prevention of the dissemination and management of Covid-19, according to general physicians and pediatricians during the initial phase of the pandemic.	Observational descriptive study	More than 80% of physicians notified patients. The waiting time for phone interviews, epidemiological investigation, and availability of isolation progressively decreased from an average of six days to 0.4 days in the 12th and 16th weeks of 2020, respectively. The cumulative weekly notification rate of new cases ranged from 3.54 to 6.84 cases per 1,000 inhabitants in the 12th and 16th weeks, respectively. From the epidemiological investigation of 1,471 probable cases, 2,514 close contacts were identified and, in turn, quarantined at home.
Duarte RB et al.^43^, 2020, Brazil	Assessing the actions of nurses working in the *Estratégia Saúde da Família* (ESF – Family Health Strategy) with regard to the role that community health agents played along with the population during the Covid-19 pandemic.	Experience report	The role of community health agents is to be a mediator between health teams and the population, developing guidance actions on the functioning of preventive services and self-care related to Covid-19 in the territories where they work. Nurses also play an important role in the training of community health agents regarding the reorganization of the work process during the pandemic and the appropriate use of personal protective equipment.
Ximenes Neto FRG et al.^44^, 2020, Brazil	Assessing strategic actions of care coordination, monitoring, and surveillance of Covid-19 cases in PHC.	Experience report	This study highlighted the importance of horizontal social isolation and home isolation of positive cases, the use of digital technologies to disseminate actions on the prevention of Covid-19 and implementation of telemedicine, the strengthening of intersectoral actions among health, education, and social assistance, and the structuring of the *Rede de Atenção à Saúde* (RAS – Health Care Network).
Coma E et al.^46^, 2020, Spain	Analyzing the effect of the Covid-19 epidemic and lockdown measures based on health care quality indicators and the control of chronic diseases.	Retrospective descriptive study	In total, 34 quality indicators were evaluated and 85% and 68% of them showed negative results in March and April 2020, respectively, when compared with the same period in 2019. Regarding treatment, monitoring, and chronic disease indicators, 100%, 80%, and 90% of them presented negative effects, respectively.
Garg S et al.^47^, 2020, India	Determining the preparation of PHC units for the provision of safe outpatient services during the Covid-19 pandemic in India.	Cross-sectional study	This study assessed 51 PHC services and identified problems in infrastructure and infection control. Care for chronic non-communicable diseases, immunization, prenatal care, and maternal and child health were the most affected areas. On the other hand, in screening sites for flu symptoms, the number of appointments increased.
Danhieux K et al.^48^, 2020, Belgium	Evaluating how PHC services aimed at chronic conditions was affected during the pandemic in Belgium.	Qualitative research	The health care organization changed, starting to focus on suspected Covid-19 cases and the use of telemedicine, and decreasing the provision of care for chronic conditions. Most professionals interviewed did not perform risk stratification and active search for patients at higher risk—telemedicine was used to evaluate and prescribe medicines and not to monitor chronic conditions. The provision of care for chronic conditions was sharply discontinued.

PHC: primary health care.

The articles studied presented a global picture of how PHC professionals and services organized themselves to tackle the Covid-19 pandemic, especially by the use of technologies and telemedicine. In total, 17 (71%) articles addressed this alternative modality of care and the monitoring of cases by applications, phone, and online platforms (Box 2).

Moreover, 13 (54%) articles addressed adjustments in infrastructure and care flows, such as the adoption of specific Covid-19 care units, the separation of infected and non-infected patients in health units, and changes in the work process and infrastructure (Box 2).

Three (12.5%) articles studied the importance of health education on Covid-19 for the community and health teams and three (12.5%) analyzed the care of chronic patients during the pandemic. Two (8.5%) articles assessed the organization of PHC services and professionals based on previous experiences, such as the H1N1 pandemic (Box 2).

## DISCUSSION

The 24 articles selected in this study addressed the reorganization of PHC services and professionals and the surveillance in the monitoring of Covid-19 cases for the care of suspected or confirmed cases.

In March 2020, after the declaration of community transmission of this disease throughout Brazil, the Secretariat of Health Surveillance of the Ministry of Health adapted the *Sistema de Vigilância de Síndromes Respiratórias Agudas* (SRAG *–* Acute Respiratory Syndromes Surveillance System), aiming to guide the *Sistema Nacional de Vigilância em Saúde* (SNVS – National Health Surveillance System) during the simultaneous circulation of SARS-CoV-2, influenza, and other respiratory viruses, within the scope of the *Emergência em Saúde Pública de Importância Nacional* (ESPIN – Public Health Emergency of National Importance)^12^.

This context shows the importance of the role of health surveillance in the notification, investigation, and monitoring of severe and confirmed Covid-19 cases^13^. Health surveillance actions are essential for the PHC organization in this new scenario, by the development of actions that enable the early identification of suspected cases, immediate notification, active search for contacts, reinforcement of home isolation, health education, and support to vulnerable groups in Brazil^14^.

The Covid-19 pandemic increased the demand for care in the SUS, which made the Brazilian Ministry of Health reinforce the need for organization of the care network and care flows both for people with flu-like diseases, including Covid-19, and who needed follow-up for other health conditions^12^.

Thus, during the Covid-19 pandemic in Brazil, the main strategy of PHC to tackle this disease was reorganizing the work process of health professionals, especially by health education activities, as it builds knowledge to provide self-care^15^. All countries had to reorganize and strengthen the responsiveness of PHC services, according to their public policies, and ensure care for other health demands of the population^16^.

Moreover, Brazil constantly updated epidemiological surveillance guides to direct the fight against Covid-19^12^. The Brazilian Ministry of Health published the *Protocolo de Manejo Clínico do Coronavírus* (Covid-19) *na Atenção Primária à Saúde* (Covid-19 Clinical Management in Primary Health Care Protocol), recommending the reorganization of PHC services and the work process to tackle the pandemic. This protocol established measures to prevent infection in health services, models for stratification of the severity of suspected cases, actions for therapeutic follow-up and home isolation of mild cases, measures for stabilization and referral to services of greater complexity, and actions to promote community prevention measures^17^.

The reorganization of the physical structure of PHC services was one of the main strategies to reduce risks of infection in health units, speed up services—avoiding contact between patients with and without suspected Covid-19—and protect professionals, maximizing the efficiency of the services provided.

Studies performed in Brazil, Belgium, Iceland, and the USA adapted health units to maintain physical distancing, adopted the immediate identification of symptomatic cases at reception, limited the number of companions, separated rooms for symptomatic and asymptomatic patients, and reorganized the care of priority groups assisted by the service^18^.

In Diadema, SP, the health management decentralized the care to respiratory symptomatic patients in all PHC units, considering the capillarity of PHC with family health teams. Thus, all services at this level of care adopted this demand^24^.

In Florianópolis, SC, Sobral, CE, and Belo Horizonte, MG, health units with adequate physical space also organized care flows separately: they treated suspected and confirmed Covid-19 patients in different places from other patients^25^.

On the other hand, one or more specific health units centralized the care of respiratory symptomatic patients. The organization of these services presented similarities, such as the respect for the instructions to patients on personal hygiene care and use of masks and the redesign of physical spaces to respect physical distancing^28^.

England created specific care units for Covid-19 patients and those who were unable to go to a health unit or did not need hospital care were monitored at home^30^.

In Rochester, Minnesota, USA, this same strategy was adopted to avoid contact between confirmed or suspected Covid-19 patients and other patients. Of the five health units in this city, one was adjusted to exclusively care for Covid-19 patients or people with symptoms of respiratory problems. Initially, they adopted phone screening to evaluate if patients needed hospital care or could be cared at the Covid-19 unit^28^.

Considering the need to adjust face-to-face care in health units in the pandemic context, strategies for remote care emerged as an alternative to monitor both Covid-19 and other patients^6,14,24,31^. Thus, telemedicine and remote monitoring were included in the routine of health professionals, aiming to decrease the number of patients in services and refer them to health units only when necessary. These measures aimed to reduce the circulation of SARS-CoV-2^20^.

In Australia, PHC started using telemedicine and call centers to screen people with respiratory symptoms, as well as developing a national network of complementary respiratory units. Moreover, health professionals participated in online trainings and health protection measures for Aboriginal communities and the population of the Torres Strait islands were disclosed^42^.

In the United Kingdom, the number of remote appointments doubled while face-to-face appointments and home visits decreased. In total, three-quarters of PHC patients were remotely cared^30,38^. In Iceland, the number of phone or online appointments increased by 127% and the number of remote medicine prescriptions and appointments also increased^18^. France, Italy, and Belgium also presented this increasing trend, since most general physicians adapted their activities to the remote modality^19,29,40^.

In Italy, the monitoring and follow-up of Covid-19 patients was performed remotely, by social networking applications, and twice a day, including the monitoring of the vital signs of patients, which were measured by smartphones. In cases of difficulty to monitor vital signs by technological devices, patients could borrow a pulse oximeter to check their saturation and heart rate at home^40^.

Although telemedicine allows the continuity of care remotely, this change can hinder the practice of general physicians due to the loss of non-verbal communication, the limited capacity of some patients to articulate their needs, and the association between intercultural communication and language problems, which are barriers in care^34^.

A study performed with the older adults cared by PHC in the United Kingdom showed that health professionals must be aware that remote appointments, especially in the case of video calls, may represent an additional barrier in the care of vulnerable groups, who have limited access to the Internet, smartphones, and other technologies^38^.

The Covid-19 pandemic promoted innovation in the care provided by PHC, since health units had to adapt themselves to physical distancing measures. However, PHC must evaluate the efficiency of remote care and identify the possible difficulties of a group in the use of the necessary technologies.

In the scope of PHC, education actions became stronger in the care provided by health professionals to limit the spread of Covid-19^22,23,32,34,43,44^. We highlight the difficulty of dealing with community rumors and misleading information, which directly affected the care process during the pandemic. Thus, integrating technology support and disseminating reliable guidelines is important^32^.

Adherence to prevention measures against Covid-19 is related to health education actions carried out by health professionals. In this sense, digital technologies are important to disseminate information about the prevention of this disease by social networks. They also reinforce the fundamental role of community health agents in health education, especially in the fight against fake news and the mediation between PHC and the community^22,43^.

In a Brazilian municipality, family health teams, along with community agents, carried out health education activities for the population. These actions aimed to guide the population on preventive measures against Covid-19 and disseminate epidemiological data by radio, sound cars, flyers, social networks, and phone. However, the number of suspected Covid-19 cases did not decrease, showing the importance of people’s adherence to the prevention measures^19^. For health education actions to achieve their objectives, developing different strategies to overcome social and cultural barriers that influence the choices of individuals is necessary^22,45^.

During the Covid-19 pandemic, the follow-up of patients with chronic non-communicable diseases (NCDs) was postponed and its number decreased worldwide, since the care of part of this cases was not urgent^21,22,29,32,34,46^. In Muscat, Oman, all health units suspended face-to-face care of patients with NCDs at the beginning of the pandemic^32^. In Spain and Belgium, although this suspension did not occur, the number of follow-ups of patients with diabetes mellitus and systemic arterial hypertension significantly decreased^46,48^.

The administration of vaccines in children reduced in Spain^46^ and outpatient services related to maternal and child health were discontinued in India^47^. France maintained outpatient care for other health problems while the Covid-19 care was directed and concentrated in specific centers^16^.

The postponement of the care of patients with NCDs, childcare, and the reduced vaccination coverage may have consequences that will extend after the Covid-19 pandemic, causing an overload in health systems^21,29,34,46^. On the other hand, the reduced attendance of patients with NCDs to health units is probably related to the health authorities’ recommendations for people to stay at home and seek these services only if in case of Covid-19 symptoms^29^.

Although health professionals working in PHC quickly organized themselves in response to the beginning of the pandemic, the targeting of Covid-19 care can cause health complications for a part of the population, whose care was postponed or suspended, and burden the health system.

Thus, the guarantee of comprehensive care during the pandemic became a major challenge for PHC, due to the valorization of care in hospital services and the detriment of other needs of the population^5,22,43,45^. Therefore, the pandemic reinforces the need to strengthen the role of PHC in the organization of health services in the SUS, as a way to optimize expenses and reduce hospitalizations, both for Covid-19 and other causes related to this level of care.

In Brazil, the Ministry of Health published guides and ordinances to guide PHC services and professionals. Moreover, state health departments, along with the *Conselho Nacional de Secretários de Saúde* (CONASS – National Council of Health Secretaries), supported municipal managers when discussing about restructuring services; however, despite of regulations, each municipality adapted itself according to its local reality and epidemiological, political, and financial issues.

PHC works as an organizer in the health network, which increases its importance in the fight against the Covid-19 pandemic, as it manages the early identification of suspected cases, the monitoring of mild cases, and the identification and referral of severe cases, besides contributing to reduce the burden of specialized and hospital services, which, consequently, reduces public spending.

This study presents an overview of how PHC services and professionals organized themselves to tackle the Covid-19 pandemic, addressing adjustments in infrastructure and care flows, such as establishing specific Covid-19 care units, separating infected and non-infected patients, using telemedicine as an alternative modality of care, and monitoring cases by applications and phone. However, gaps still exist in the literature, such as the evaluation of the effect of these actions and their effectiveness in mitigating the Covid-19 transmission, the analysis of the consequences for patients whose care was postponed or reduced (patients with NCDs, childcare follow-up, prenatal care, and vaccination coverage, for example) during the pandemic.

## References

[1] World Health Organization (2021). Coronavirus disease (COVID-19) pandemic.

[2] Ministério da Saúde (BR) (2022). Painel de casos de doença pelo coronavírus 2019 (COVID-19) no Brasil pelo Ministério da Saúde.

[3] Tabish SA (2020). COVID-19 pandemic: emerging perspectives and future trends. J Public Health Res.

[4] World Health Organization, Regional Office for the Western Pacific Region (2021). Role of primary care in the COVID-19 Response: interim guidance.

[5] Alves MTG (2020). Reflexões sobre o papel da Atenção Primária à Saúde na pandemia de COVID-19. Rev Bras Med Fam Comunidade.

[6] Cabral ERM, Bonfada D, Melo MC, Cesar ID, Oliveira REM, Bastos TF (2020). Contributions and challenges of the Primary Health Care across the pandemic COVID-19. InterAm J Med Health.

[7] Sarti TD, Lazarini WS, Fontenelle LF, Almeida APSC (2020). What is the role of Primary Health Care in the COVID-19 pandemic?. Epidemiol Serv Saude.

[8] Conselho Nacional de Secretários de Saúde (2020). Guia orientador para o enfrentamento da pandemia COVID-19 na Rede de Atenção à Saúde.

[9] Aromataris E, Munn Z (2020). JBI manual for evidence synthesis.

[10] Fabbri S, Silva C, Hernandes E, Octaviano F, Di Thommazo A, Belgamo A (2016). Improvements in the StArt tool to better support the systematic review process.

[11] Tricco AC, Lillie E, Zarin W, O’Brien KK, Colquhoun H, Levac D (2018). PRISMA Extension for Scoping Reviews (PRISMA-ScR): checklist and explanation. Ann Intern Med.

[12] Ministério da Saúde (BR), Secretaria de Vigilância em Saúde (2021). Guia de vigilância epidemiológica: emergência de saúde pública de importância nacional pela doença pelo coronavírus 2019 – COVID-19.

[13] Jesus AM, Oliveira KNR, Silva MPF, Matos RB, Dias CFMA (2021). Rede de vigilância no monitoramento da Covid-19 na Bahia, Brasil, 2020. Rev Baiana Saude Publica.

[14] Giovanella L, Martufi V, Mendoza DCR, Mendonça MHM, Bousquat AEM, Aquino R (2020). A contribuição da Atenção Primária à Saúde na rede SUS de enfrentamento à Covid-19. Saude Debate.

[15] Geraldo SM, Farias SJM, Sousa FOS (2021). A atuação da Atenção Primária no contexto da pandemia da COVID-19 no Brasil. Res Soc Dev.

[16] Prado NMBL, Rossi TRA, Chaves SCL, Barros SG, Magno L, Santos HLPC (2020). The international response of primary health care to COVID-19: document analysis in selected countries. Cad Saude Publica.

[17] Ministério da Saúde (BR), Secretaria de Atenção Primária à Saúde (2020). Protocolo de manejo clínico do coronavírus (COVID-19) na Atenção Primária à Saúde - Versão 9.

[18] Krist AH, DeVoe JE, Cheng A, Ehrlich T, Jones SM (2020). Redesigning primary care to address the COVID-19 pandemic in the midst of the pandemic. Ann Fam Med.

[19] Morreel S, Philips H, Verhoeven V (2020). Organisation and characteristics of out-of-hours primary care during a COVID-19 outbreak: a real-time observational study. PLoS One.

[20] Fernandes LMM, Pacheco RA, Fernandez M (2020). How a Primary Health Care Clinic in Brazil faces coronavirus treatment within a vulnerable community: the experience of the Morro da Conceição area in Recife. NEJM Catal Innov Care Deliv.

[21] Sigurdsson EL, Blondal AB, Jonsson JS, Tomasdottir MO, Hrafnkelsson H, Linnet K (2020). How primary healthcare in Iceland swiftly changed its strategy in response to the COVID-19 pandemic. BMJ Open.

[22] Dias EG, Ribeiro DRSV (2020). Manejo do cuidado e a educação em saúde na atenção básica na pandemia do Coronavírus. J Nurs Health.

[23] Vieira DS, Sá PC, Torres RC, Oliveira FT, Rocha KRSL, Vasconcelos TLC (2020). Planejamento da enfermagem frente à covid-19 numa estratégia de saúde da família: relato de experiência. Saude Coletiva (Barueri).

[24] Cirino FMSB, Aragão JB, Meyer G, Campos DS, Gryschek ALFPL, Nichiata LYI (2021). Desafios da atenção primária no contexto da COVID-19: a experiência de Diadema, SP. Rev Bras Med Fam Comunidade.

[25] Silveira JPM, Zonta R (2020). Experiência de reorganização da APS para o enfrentamento da COVID-19 em Florianópolis. APS Rev.

[26] Ribeiro MA, Araújo DG, Cavalcante ASP, Martins AF, Sousa LA, Carvalho RC, Cunha ICKO (2020). (RE)Organização da Atenção Primária à Saúde para o enfrentamento da COVID-19: experiência de Sobral-CE. APS Rev.

[27] Guimarães FG, Carvalho TML, Bernardes RM (2020). A organização da Atenção Primária à Saúde de Belo Horizonte no enfrentamento da pandemia Covid 19: relato de experiência. APS Rev.

[28] Jacobson NA, Nagaraju D, Miller JM, Bernard ME (2020). COVID Care Clinic: a unique way for family medicine to care for the community during the SARS-CoV-2 (COVID-19) pandemic. J Prim Care Community Health.

[29] Saint-Lary O, Gautier S, Le Breton J, Gilberg S, Frappé P, Schuers M (2020). How GPs adapted their practices and organisations at the beginning of COVID-19 outbreak: a French national observational survey. BMJ Open.

[30] Majeed A, Maile EJ, Bindman AB (2020). The primary care response to COVID-19 in England’s National Health Service. J R Soc Med.

[31] Engstrom E, Melo E, Giovanella L, Mendes A, Grabois V, Mendonça MHM (2020). Recomendações para a organização da Atenção Primária à Saúde no SUS no enfrentamento da COVID-19.

[32] Al Ghafri T, Al Ajmi F, Anwar H, Al Balushi L, Al Balushi Z, Al Fahdi F (2020). The experiences and perceptions of health-care workers during the COVID-19 pandemic in Muscat, Oman: a qualitative study. J Prim Care Community Health.

[33] Blazey-Martin D, Barnhart E, Gillis J, Vazquez GA (2020). Primary care population management for COVID-19 patients. J Gen Intern Med.

[34] Verhoeven V, Tsakitzidis G, Philips H, Van Royen P (2020). Impact of the COVID-19 pandemic on the core functions of primary care: will the cure be worse than the disease? A qualitative interview study in Flemish GPs. BMJ Open.

[35] Karim SI, Irfan F, Batais MA (2020). Becoming virtual: a preliminary experience of outpatient primary care during COVID-19 pandemic. Pan Afr Med J.

[36] Motlhatlhedi K, Bogatsu Y, Maotwe K, Tsima B (2020). Coronavirus disease 2019 in Botswana: contributions from family physicians. Afr J Prim Health Care Fam Med.

[37] Sinha S, Kern LM, Gingras LF, Reshetnyak E, Tung J, Pelzman F (2020). Implementation of video visits during COVID-19: lessons learned from a primary care practice in New York City. Front Public Health.

[38] Joy M, McGagh D, Jones N, Liyanage H, Sherlock J, Parimalanathan V (2020). Reorganisation of primary care for older adults during COVID-19: a cross-sectional database study in the UK. Br J Gen Pract.

[39] Al Ghafri T, Al Ajmi F, Al Balushi L, Kurup PM, Al Ghamari A, Al Balushi Z (2021). Responses to the pandemic COVID-19 in Primary Health Care in Oman: Muscat experience. Oman Med J.

[40] Bressy S, Zingarelli EM (2020). Technological devices in COVID-19 primary care management: the Italian experience. Fam Pract.

[41] Mantovani W, Franchini S, Mazzurana M, Zuccali MG, Pizzo F, Zanin A (2020). Reorganization and public health management by the Department of Prevention during the COVID-19 emergency. An experience of integration between prevention and primary care in the proactive management of possible cases. Epidemiol Prev.

[42] Desborough J, Dykgraaf SH, Toca L, Davis S, Roberts L, Kelaher C (2020). Australia’s national COVID-19 primary care response. Med J Aust.

[43] Duarte RB, Medeiros LMF, Araújo MJAM, Cavalcante ASP, Souza EC, Alencar OM (2020). Agentes Comunitários de Saúde frente à COVID-19: vivências junto aos profissionais de enfermagem. Enferm Foco.

[44] Ximenes FRG, Araújo CRC, Silva RCC, Ribeiro MA, Sousa LA, Serafim TF (2020). Coordenação do cuidado, vigilância e monitoramento de casos da COVID-19 na Atenção Primária à Saúde. Enferm Foco.

[45] Rios AFM, Lira LSSP, Reis IM, Silva GA (2020). Atenção Primária à Saúde frente à COVID-19: relato de experiência de um Centro de Saúde. Enferm Foco.

[46] Coma E, Mora E, Méndez L, Benítez M, Hermosilla E, Fàbregas M (2020). Primary care in the time of COVID-19: monitoring the effect of the pandemic and the lockdown measures on 34 quality of care indicators calculated for 288 primary care practices covering about 6 million people in Catalonia. BMC Fam Pract.

[47] Garg S, Basu S, Rustagi R, Borle A (2020). Primary Health Care facility preparedness for outpatient service provision during the COVID-19 pandemic in India: cross-sectional study. JMIR Public Health Surveill.

[48] Danhieux K, Buffel V, Pairon A, Benkheil A, Remmen R, Wouters E (2020). The impact of COVID-19 on chronic care according to providers: a qualitative study among primary care practices in Belgium. BMC Fam Pract.

