# Supplementary material for: Primary health care organization in the Covid-19 pandemic: scoping review
Source: Rev Saude Publica. 2022 Nov 3;56:94. doi: 10.11606/s1518-8787.2022056004374 (PMC9635848; doi:10.11606/s1518-8787.2022056004374)
Supplement: Translation

# Translation: Organização da atenção primária à saúde na pandemia de covid-19: revisão de escopo

**Authors:** Breno Ribeiro Gonçalves da Silva, Ana Paula de Vechi Corrêa, Sílvia Carla da Silva André Uehara

**Affiliations:** I Brasil Universidade Federal de São Carlos. Centro de Ciências Biológicas e da Saúde. Departamento de Enfermagem. Graduação em Enfermagem. São Carlos, SP, Brasil; II Brasil Universidade Federal de São Carlos. Centro de Ciências Biológicas e da Saúde. Departamento de Enfermagem. Programa de Pós-Graduação em Enfermagem. São Carlos, SP, Brasil; III Brasil Universidade Federal de São Carlos. Centro de Ciências Biológicas e da Saúde. Departamento de Enfermagem. São Carlos, SP, Brasil

**Keywords:** Atenção Primária à Saúde, organização & administração, COVID-19, prevenção & controle, Capacitação de Recursos Humanos em Saúde, Revisão

## Abstract

**OBJETIVO:**

Mapear evidências científicas disponíveis sobre a organização dos serviços e profissionais da atenção primária à saúde durante o enfrentamento da covid-19.

**MÉTODO:**

Trata-se de uma revisão de escopo, seguindo o método do Joanna Briggs Institute. Foram incluídos estudos publicados em português, espanhol e inglês, no período de janeiro/2020 a janeiro/2021, nas bases de dados CINAHL, Lilacs, Medline, PubMed e Web of Science.

**RESULTADOS:**

Foram selecionados 24 artigos, que mostraram a reorganização dos serviços e profissionais da atenção primária à saúde para o atendimento de casos suspeitos ou confirmados de covid-19. As ações de coordenação do enfrentamento da doença na atenção primária à saúde colaboram no controle da infecção, especialmente por meio de busca ativa de sintomáticos respiratórios e detecção de casos novos, seguido do monitoramento dos casos confirmados.

**CONCLUSÕES:**

O presente estudo mostra um panorama de como profissionais e serviços da atenção primária se organizaram no enfrentamento da pandemia, abordando as readequações adotadas na infraestrutura e fluxos de atendimentos, a exemplo da adoção de unidades específicas no atendimento para covid-19, separação de fluxos infectado/não infectado, telemedicina como alternativa de modalidade de atendimento e monitoramento dos casos por meio de aplicativos e telefone.

## INTRODUÇÃO

Enfrentar a pandemia causada pelo novo coronavírus exigiu que os sistemas de saúde de todo o mundo fossem remodelados, de modo que houvesse diferentes respostas no enfrentamento da infecção, a fim de impedir a sua disseminação e reduzir as sequelas causadas pela doença na população. Isso se deve, principalmente, à elevada taxa de transmissibilidade da covid-19, uma vez que a doença se espalhou rapidamente pelo mundo, resultando, desde o início da pandemia até o primeiro trimestre de 2022, em mais de 500 milhões de casos confirmados e mais de seis milhões de óbitos em todo o mundo^1^. No mesmo período, o Brasil apresentou mais de 30 milhões de casos confirmados e o número de óbitos acumulados já ultrapassou 660 mil^2^.

O contexto pandêmico desencadeou desafios para os sistemas de saúde, evidenciando as fragilidades a partir da exposição de problemas crônicos de financiamento e de gestão^3^. Ressalta-se a importância da preparação desses sistemas para o enfrentamento de doenças infecciosas emergentes, por meio de planejamento e estratégias embasadas em evidências, com coordenação entre todos os segmentos de um sistema e o governo.

Nessa perspectiva, no Brasil, a atenção primária à saúde (APS) se destaca, uma vez que está geralmente estruturada como a principal porta de entrada dos serviços de saúde no âmbito do Sistema Único de Saúde (SUS). A APS está organizada de forma a proporcionar a resolução da maior parte dos problemas de saúde dos indivíduos e de suas famílias, desempenhando um papel fundamental no enfrentamento da pandemia, uma vez que grande parte das pessoas infectadas desenvolvem a forma leve da doença, o que permite o seu acompanhamento nesse nível da assistência^4^.

Considerando as ações que devem ser desenvolvidas no enfrentamento da covid-19, em especial na atenção primária, o conhecimento do território, o acesso, o vínculo entre o usuário e a equipe de saúde, a integralidade da assistência, o monitoramento das famílias vulneráveis e o acompanhamento de casos suspeitos e leve da doença, proporciona a elaboração de estratégias fundamentais para a contenção da pandemia, bem como para o não agravamento da doença^7^.

No Brasil, a APS segue como ponto central na organização do SUS, comprovando a necessidade de fortalecê-la e organizá-la para que fosse o pilar do enfretamento da covid-19, devido a sua capacidade de vínculo, manejo e acompanhamento dos casos no momento da pandemia e para a retomada de suas rotinas no pós-pandemia, olhando para o contexto do território como um todo^7,8^.

Assim, considerando a pandemia de covid-19, enfatiza-se o papel fundamental da atenção primária no enfrentamento global à doença, desenvolvendo ações educativas, preventivas, promotoras da saúde, assistenciais e administrativas. A organização da APS para a oferta de serviços efetivos é responsabilidade de todos os profissionais de saúde e gestores, sendo desenvolvidos não somente nas unidades de saúde, mas em todo território e domicílio.

Estudos têm sido publicados sobre a evolução da doença, tratamento, vacina, entretanto, torna-se necessário o mapeamento das evidências científicas sobre estudos que investigam a organização dos serviços e profissionais de saúde da APS e o impacto no enfrentamento da pandemia.

Desse modo, esta revisão permite mostrar as lacunas persistentes sobre a temática, além de refletir sobre a organização das ações desse nível de assistência para a mitigação da doença e, assim, se poder planejar estratégias para o enfrentamento da covid-19, de modo a proporcionar uma assistência resolutiva e de qualidade à população assistida. Logo, este estudo tem como objetivo mapear as evidências científicas disponíveis sobre a organização dos serviços e dos profissionais de saúde na atenção primária à saúde durante o enfrentamento da covid-19.

## MÉTODOS

Trata-se de um estudo de *scoping review* com base nos princípios apresentados pelo *Joanna Briggs Institute* (JBI): (1) identificação da questão de pesquisa, (2) identificação de estudos relevantes, (3) seleção dos estudos, (4) extração de dados, (5) separação, sumarização e relatório de resultados e (6) divulgação dos resultados^9^.

Para realizar a estratégia de busca da revisão, foi utilizada a proposta do JBI, que consiste em um acrônimo “PCC”, que representa “P” população, “C” conceito e “C” contexto. A questão norteadora do estudo foi construída com base na estratégia PCC, sendo “P” (profissionais de saúde), “C” (atenção primária à saúde) e “C” (organização e covid-19), definida da seguinte forma: Como foi a organização dos serviços e dos profissionais de saúde da atenção primária à saúde durante a pandemia de covid-19?

A busca dos artigos foi realizada nas bases de dados eletrônicas: *US National Library of Medicine National Institutes of Health* (PubMed), *Medical Literature Analysis and Retrieval System Online* (Medline), *Institute for Scientific Information* (Web of Science), Literatura Latino-Americana e do Caribe em Ciências da Saúde (Lilacs) e *Cumulative Index to Nursing and Allied Health Literature* (CINAHL). As buscas foram realizadas entre os meses de março e maio de 2021 e conduzidas por meio de descritores e seus sinônimos que constam no Descritor em Ciências da Saúde (DeCS) e no *Medical Subject Headings* (MeSH), nos diferentes idiomas, sendo eles: organização, assistência, atenção primária à saúde, covid-19, profissionais de saúde (Quadro 1).

**Quadro 1:** Estratégias de busca utilizadas nas bases de dados.

Base de dados	Estratégias de busca
PubMed	(((covid-19[Title]) OR coronavirus[Title])) AND (primary health care[Title] OR primary care[Title])) AND organization[Body - All Words]; (((covid-19[Title]) OR coronavirus[Title])) AND (primary health care[Title] OR primary care[Title])) AND assistance[Body - All Words]; (((covid-19[Title] OR coronavirus[Title])) AND (primary health care[Abstract] OR primary care[Abstract])) AND assistance[Body - All Words]; (((covid-19[Title] OR coronavirus[Title])) AND (primary health care[Abstract] OR primary care[Abstract])) AND organization[Body - All Words]; ((((covid-19[Title] OR coronavirus[Title])) AND (primary health care[Abstract] OR primary care[Abstract])) AND healthcare professionals[Abstract]) AND organization[Body - All Words]; (((covid-19[Title] OR coronavirus[Title])) AND (Family Health Strategy[Abstract] OR family health[Abstract])) AND organization[Body - All Words].
Medline	TI (covid-19 OR coronavirus OR coronavírus) AND AB (primary health care OR primary care OR atenção primária OR primeros auxilios) AND AB (organization OR organização OR organización); TI (covid-19 OR coronavirus OR coronavírus) AND TI (primary health care OR primary care OR atenção primária OR primeros auxilios) AND AB (organization OR organização OR organización); TI (covid-19 OR coronavirus OR coronavírus) AND AB (primary health care OR primary care OR atenção primária OR primeros auxilios) AND AB (assistance OR assistência OR asistencia); TI (covid-19 OR coronavirus OR coronavírus) AND AB (primary health care OR primary care OR atenção primária OR primeros auxilios) AND AB (organization OR organização OR organización) AND AB (healthcare professionals OR profesionales de la salud OR profissionais de saúde OR profissionais OR profesionales OR professionals); TI (covid-19 OR coronavirus OR coronavírus) AND AB (Family Health Strategy OR family health OR Estratégia Saúde da Família OR Saúde da Família OR salud familiar) AND AB (organization OR organização OR organización).
Web of Science	TÍTULO: (covid-19 OR coronavirus OR coronavírus) AND TÍTULO: (primary health care OR primary care OR atenção primária OR primeros auxilios) AND TÓPICO: (organization OR organização OR organización); TÍTULO: (covid-19 OR coronavirus OR coronavírus) AND TÓPICO: (primary health care OR primary care OR atenção primária OR primeros auxilios) AND TÓPICO: (organization OR organização OR organización); TÍTULO: (covid-19 OR coronavirus OR coronavírus) AND TÓPICO: (primary health care OR primary care OR atenção primária OR primeros auxilios) AND TÓPICO: (assistance OR assistência OR asistencia); TÍTULO: (covid-19 OR coronavirus OR coronavírus) AND TÓPICO: (primary health care OR primary care OR atenção primária OR primeros auxilios) AND TÓPICO: (organization OR organização OR organización) AND TÓPICO: (healthcare professionals OR profesionales de la salud OR profissionais de saúde OR profissionais OR profesionales OR professionals); TÍTULO: (covid-19 OR coronavirus OR coronavírus) AND TÓPICO: (Family Health Strategy OR family health OR Estratégia Saúde da Família OR Saúde da Família OR salud familiar) AND TÓPICO: (organization OR organização OR organización).
Lilacs	covid-19 OR coronavirus OR coronavírus [Title words] and primary health care OR primary care OR atenção primária OR primeros auxilios [Words] and organization OR organização OR organización [Words]; covid-19 OR coronavirus OR coronavírus [Palavras do título] and primary health care OR primary care OR atenção primária OR primeros auxilios [Palavras] and assistance OR assistência OR asistencia [Palavras]; covid-19 OR coronavirus OR coronavírus [Palavras do título] and primary health care OR primary care OR atenção primária OR primeros auxilios [Palavras] and healthcare professionals OR profesionales de la salud OR profissionais de saúde OR profissionais OR profesionales OR professionals [Palavras].
CINAHL	TI (covid-19 OR coronavirus OR coronavírus) AND AB (primary health care OR primary care OR atenção primária OR primeros auxilios) AND AB (organization OR organização OR organización); TI (covid-19 OR coronavirus OR coronavírus) AND AB (primary health care OR primary care OR atenção primária OR primeros auxilios) AND AB (organization OR organização OR organización) AND AB (healthcare professionals OR profesionales de la salud OR profissionais de saúde OR profissionais OR profesionales OR professionals); TI ( covid-19 OR coronavirus OR coronavírus ) AND AB ( Family Health Strategy OR family health OR Estratégia Saúde da Família OR Saúde da Família OR salud familiar ) AND AB ( organization OR organização OR organización ); TI ( covid-19 OR coronavirus OR coronavírus ) AND AB ( primary health care OR primary care OR atenção primária OR primeros auxilios ) AND AB ( organization OR organização OR organización ) AND AB ( nursing OR nurse OR nursing care OR nursing practice OR enfermagem OR enfermería ).

Foram aplicados os seguintes critérios de inclusão: estudos primários, idiomas português, inglês e espanhol, publicação no período de 1 de janeiro de 2020 até 31 de janeiro de 2021, indexados nas bases de dados citadas. Foram excluídos protocolos, editoriais, revisões sistemáticas e estudos cujo título e resumo não responderam à pergunta problema, informações de websites e veiculadas pela mídia.

Na etapa seguinte, as referências foram exportadas para o aplicativo *web StArt* (*State of the Art through Systematic Review*), para a seleção dos estudos em dois níveis. A primeira seleção foi por meio da leitura de títulos e resumos, seguida pela leitura do artigo na íntegra. A ferramenta de revisão *StArt* foi desenvolvida pelo Laboratório de Pesquisa em Engenharia de Software (LaPES) da Universidade Federal de São Carlos (UFSCar)^10^.

Os estudos elegíveis foram recuperados na íntegra e avaliados por dois pesquisadores. Em ambas as fases, as divergências foram discutidas até chegar a um consenso e, então, realizar a seleção final, com a participação de um terceiro pesquisador. O relato do estudo foi pautado no *Preferred Reporting Items for Systematic Reviews and Meta-analyses Extension for Scoping Reviews* (PRISMA_ScR)^11^.

As informações relevantes foram coletadas de cada artigo selecionado: autores, nome do periódico, país onde o estudo foi realizado, país de publicação, desenho do estudo, principais resultados e lacunas. Os resultados revisados estão apresentados em quadros e no formato narrativo sobre aspectos bibliométricos e que respondem à questão que orientou esta revisão de escopo.

## RESULTADOS

Foram identificados 1.262 artigos nas bases de dados, desses, foram excluídos 374 por serem duplicatas, 833 após a leitura dos títulos e resumos, e 31, após a leitura na íntegra. Assim, foram selecionados para o estudo 24 artigos que abordavam a organização dos serviços e dos profissionais de saúde da APS durante o enfrentamento da covid-19 (Figura).

**Figura:** Fluxograma de referência: inclusão e exclusão dos artigos.

Os estudos incluídos nesta revisão foram publicados entre 1 de janeiro de 2020 e 31 de janeiro de 2021, sendo que cinco (21%) foram realizados no Brasil; quatro (17%) nos Estados Unidos; dois (8%) em Omã, Reino Unido, Itália e Bélgica; e um (4%) na Espanha, Índia, Flandres, Arábia Saudita, Botsuana, França e Islândia. Quanto aos idiomas, 20 (83%) estudos foram publicados em inglês e quatro (17%) em português.

Dentre os 24 estudos que foram selecionados para análise, destacam-se nove relatos de experiência, cinco estudos observacionais, quatro descritivos, dois transversais, dois qualitativos e dois estudos de caso (Quadro 2).

**Quadro 2:** Artigos selecionados na revisão, segundo local e ano do estudo, objetivo, tipo de estudo, principais resultados.

Autor, ano e local	Objetivo	Tipo de estudo	Principais resultados
Krist AH et al.^18^, 2020, Estados Unidos	Apresentar ações a serem adotadas pela atenção primária durante a pandemia de acordo com protocolo do Centro de Controle e Prevenção de Doenças (CDC).	Estudo descritivo	O enfrentamento da pandemia de covid-19 na atenção primária foi organizado em 6 fases: 1) vigilância e notificação e monitoramento dos casos; 2) distanciamento físico, aumentar as consultas virtuais e adiar consultas não urgentes; 3) implantação de ações para achatar a curva epidemiológica; 4) encaminhamento aos hospitais somente de casos graves; 5) ações de cuidados de pacientes em convalescença; 6) abordar as consequências da pandemia.
Morreel S et al.^19^, 2020, Bélgica	Descrever a organização e as características das consultas na atenção primária realizadas fora do horário de expediente, e compará-las com o mesmo período em 2019.	Estudo observacional	Triagem telefônica, classificando os paciente como suspeito ou regular. Casos suspeitos de covid-19 eram todos atendidos de forma virtual e caso necessário encaminhados para unidades de atendimento exclusivo de suspeitos de covid-19 (Unidades Corona), ou visita domiciliar, ou encaminhados ao pronto socorro. Em comparação com 2019, houve aumento na carga de trabalho devido a atendimentos por telefone, porém as consultas presenciais reduziram em 45%.
Fernandes LMM et al.^20^, 2020, Brasil	Descrever a adaptação de um centro de atenção primária à saúde em Recife e aprimorou as capacidades de telessaúde e monitoramento remoto.	Relato de caso	Reorganização do fluxo interno da unidade, separando pacientes sintomáticos de assintomáticos; suspensão das atividades coletivas; manutenção do acompanhamento dos pacientes com doenças crônicas; utilização de teleatendimento e monitoramento remoto; ações de vigilância ativa no território pela equipe.
Sigurdsson EL et al.^21^, 2020 Islândia	Descrever como a atenção primária à saúde na Islândia mudou sua estratégia para lidar com a pandemia de covid-19.	Descritivo observacional	Detecção precoce de casos suspeitos; triagem eficaz; separação de fluxo de pacientes sintomáticos e assintomáticos; manutenção das atividades voltadas à maternidade e puericultura; alteração do atendimento presencial para teleatendimento; atendimento em horário alternativo. Mudança nos 10 principais diagnósticos, sendo que imunização, depressão, hipotireoidismo e lombalgia não estavam mais entre os 10 principais diagnósticos. Essas mudanças apontaram para uma APS muito sólida e com grande flexibilidade em sua organização.
Dias EG^22^, 2020, Brasil	Refletir sobre o manejo do cuidado e a educação em saúde na atenção básica no enfrentamento da pandemia do coronavírus.	Relato de experiência	Identificação imediata do sintomático respiratório; espaço reservado para aguardar a consulta; suspensão de algumas atividades assistenciais; teleatendimento e telemonitoramento; visita domiciliar ou atendimento presencial para os casos necessários; educação em saúde por meio de rádios, carros de som, panfletos, cartazes, redes sociais, contato telefônico.
Vieira DS et al.^23^, 2020, Brasil	Delinear uma experiência de planejamento organizacional da enfermagem junto à equipe de saúde da família frente à pandemia da covid-19, em uma área rural de Igreja Nova – AL.	Relato de experiência	Adotou-se três estratégias: •Orientação da comunidade sobre o problema com ações de prevenção e promoção da saúde. •Educação permanente em saúde para a equipe, para qualificação dos profissionais. •Organização da oferta dos serviços de saúde com destinação de sala exclusiva para atendimento de sintomáticos respiratórios, além de organizar os atendimentos dos programas atendidos pela unidade.
Jacobson, NA et al.^28^, 2020, Brasil	Descrever um esforço colaborativo para cuidar de uma comunidade durante a pandemia da covid-19 a partir do desenvolvimento de uma clínica.	Relato de experiência	Adaptação de uma clínicas que realizava atendimento de atenção primária para atendimento exclusivo de covid-19. Avaliação virtual, pela plataforma Zoom, realizado por enfermeiros que definiam se o paciente precisaria de atendimento de emergência ou atendimento na clínica covid. Posteriormente, o paciente tinha uma consulta presencial agendada na unidade específica. Houve readequação do espaço físico para maximizar o distanciamento social.
Saint-Lary O et al.^29^, 2020, França	Descrever como os médicos generalistas adaptaram suas práticas para garantir e manter o acesso aos cuidados na fase epidêmica.	Observacional descritivo	A partir das respostas de 5.424 médicos generalistas franceses, evidenciou-se que 70,9% mudaram o atendimento presencial para atendimento remoto; 66,5% aumentaram as consultas remotas e 42,7% criaram um fluxo específico para pacientes suspeitos de covid-19. Dos 70,9% que adaptaram sua prática, 91,7% atenderam por telefone, 27,6% por e-mail e 30,7% aumentaram o uso de consultas por videochamadas.
Majeed A et al.^30^, 2020, Inglaterra	Descrever a resposta da atenção primária ao covid-19 no Serviço Nacional de Saúde (NHS) da Inglaterra.	Observacional descritivo	Adaptação dos atendimentos presenciais para o atendimento remoto; atendimento de pacientes suspeitos ou confirmados de covid-19 eram atendidos em clinicas específicas; implantação de visitas domiciliares específicas para atendimento de covid-19; informatização completa de todas as unidades do Serviço Nacional de Saúde, usando registros médicos eletrônicos; acesso online para pacientes a serviços como marcação de consultas, pedidos de repetição de prescrições e visualização de registros médicos; envio eletrônico de receitas diretamente às farmácias de escolha do próprio paciente.
Al Ghafri T et al.^32^, 2020, Omã	Explorar as experiências e percepções dos profissionais de saúde na atenção primária à saúde na gestão da covid-19 com relação às experiências de resposta médica, reformas socioculturais e religiosas, impressões psicológicas e lições aprendidas.	Estudo qualitativo com abordagem fenomenológica empírica	Emergiram as seguintes percepções: rápida reestruturação dos serviços de APS; uso da tecnologia e desafios de se trabalhar na pandemia de covid-19; mudanças nas normas sociais e religiosas; aparecimento das lacunas no acesso aos cuidados de saúde entre os grupos vulneráveis; surgimento de distúrbios psicológicos como consequências do distanciamento social, manejo de cadáveres, exaustão dos profissionais de saúde e risco de exposição; desenvolvimento de capacidades epidemiológicas e de saúde pública, melhorando o acesso aos cuidados de saúde.
Blazey-Martin D et al.^33^, 2020, Estados Unidos	Desenvolvimento de uma abordagem inovadora de gerenciamento de população para o gerenciamento remoto de pacientes covid-19.	Relato de experiência	Foi implementado um algoritmo com o objetivo de orientar as decisões de triagem, incluindo a frequência de contato com os pacientes, dependendo do dia do início dos sintomas e dos fatores de risco; identificar achados clínicos que indicam se o paciente está seguro para permanecer em casa ou necessitam de avaliação adicional. Essas intervenções quando realizadas em tempo hábil, aliviaram a demanda dos hospitais e pronto atendimentos.
Verhoeven V et al.^34^, 2020, Flandres	Obter *insights* sobre as consequências do surto covid-19 nas competências essenciais da medicina de família e comunidade,vivenciadas por médicos na linha de frente.	Relato de experiência	Adoção de triagem e consultas por telefone, para todos os pacientes. A tomada de decisão clínica focada na avaliação e triagem respiratória; comprometimento dos cuidados agudos pela mudança do foco apenas para a covid-19 e também pelo fato dos pacientes não procurarem atendimento para os problemas que não estão relacionados à covid-19; adiamento do cuidado crônico.
Karim SI et al.^35^, 2020, Arábia Saudita	Descrever as estratégias adotas na reorganização dos atendimentos de atenção primária à saúde de um hospital da capital da Arábia Saudita, durante a pandemia de covid-19.	Relato de experiência	Os serviços foram adaptados com base em quatro pilares principais: vigilância e detecção de casos, gerenciamento clínico, prevenção da disseminação e manutenção de serviços essenciais. Essas alterações foram feitas pelo Departamento de Medicina de Família, e os atendimentos passaram a ser realizados de maneira remota por WhatsApp e clínicas virtuais e novos modos de comunicação foram incorporados como sites, mensagens do portal e mídias sociais.
Motlhatlhedi K et al.^36^, 2020, Botsuana	Explorar a ação de médicos de saúde da família durante a pandemia.	Observacional descritivo	O papel dos médicos de família foram: controle de infecções e medidas de prevenção de doenças, identificação de casos e disseminação de informações, continuidade dos cuidados médicos, especialmente para pacientes. O uso de mídia social no WhatsApp se expandiu para incluir webinars online, disseminação de informações relacionadas à covid-19 de várias fontes e do centro de coordenação nacional.
Sinha S et al.^37^, 2020, estados Unidos	Descrever a implementação e avaliação de um programa de atendimentos por vídeo em uma grande clínica acadêmica de atenção primária em Nova York.	Estudo de caso	Foram realizados 1.030 atendimentos por vídeo para 817 pacientes. 42% dos atendimentos foram para sintomas de covid-19 e os demais para condições agudas ou crônicas; os adultos jovens, mulheres e aqueles com seguro comercial, foram mais frequentes; o grau de satisfação foi alto (média de 4,6 em uma escala de 5 pontos [SD = 0,97]).
Joy M et al.^38^, 2020, Inglaterra	Avaliar a capacidade de resposta e a priorização do tipo de consulta de atenção primária para idosos durante a pandemia covid-19.	Estudo transversal	A taxa de consultas por telefone e vídeo mais do que dobrou durante o período de estudo (106,0% e 102,8%, respectivamente). As consultas presenciais diminuíram em 64,6% e as visitas domiciliares em 62,6%, esse processo coincidiu com a mudança da política nacional. O aumento relativo nas consultas esteve associado entre as pessoas que tomavam ≥ 10 medicamentos em comparação com aqueles que não tomaram nenhum.
Al Ghafri T et al.^39^, 2020, Omã	Descrever as respostas realizadas nas unidades de atenção primária à saúde de janeiro a abril de 2020, incluindo medidas de saúde pública em Muscate, Omã.	Estudo descritivo	Houve redução nas consultas, de 115.324 em janeiro para 109.719 em março. Os serviços essenciais foram garantidos em todos os centros de saúde, principalmente para grupos vulneráveis, mulheres e crianças. Os centros de saúde foram abertos por 24 horas para garantir o vigor da testagem e isolamento. Adotou-se teleatendimento e comunicações virtuais. Realização de campanhas sobre a importância do distanciamento social e higienização das mãos.
Bressy S^40^, 2020, Itália	Descrever a experiência da gestão da atenção primária no enfrentamento da covid-19 na Itália durante o início da pandemia.	Relato de experiência	A partir da experiência italiana, conclui-se que o suporte tecnológico e a abordagem remota são essenciais na avaliação de cuidados primários de covid-19. O uso da telemedicina e o auxílio da tecnologia permitiram monitorar de forma eficiente os pacientes em casa, reduzindo internações inadequadas e encaminhando para um hospital apenas quando necessário.
Mantovani W et al.^41^, 2020, Itália	Descrever a organização e o papel do Departamento de Prevenção da Unidade Local de Saúde de Trento contra disseminação e manejo da covid-19, relatados por médicos generalistas e pediatras da família durante a fase inicial da pandemia.	Descritivo observacional	Mais de 80% dos médicos notificaram pacientes. O tempo de espera para entrevista telefônica, investigação epidemiológica e disponibilização de isolamento, diminuiu progressivamente de uma média de seis dias para 0,4 dias, respectivamente, na 12ª e 16ª semana de 2020. A taxa de notificação semanal acumulada de casos novos variou de 3,54 a 6,84 casos por 1.000 habitantes, respectivamente, na 12ª e 16ª semanas. A partir da investigação epidemiológica de 1.471 casos prováveis, foram identificados 2.514 contatos próximos, e por sua vez colocados em quarentena domiciliar.
Duarte RB et al.^43^, 2020, Brasil	Descrever as ações de enfermeiras atuantes na estratégia saúde da família a respeito do papel que os agentes comunitários de saúde desenvolvem junto à população durante a pandemia da covid-19.	Relato de experiência	Evidenciou-se o papel do agente comunitário de saúde como mediador entre a equipe de saúde e a população, desenvolvendo ações de orientação nos territórios sobre o funcionamento dos serviços e autocuidados preventivos relacionados à covid-19. As enfermeiras também desempenharam um importante papel nas capacitações dos agentes comunitários de saúde a respeito da reorganização do processo de trabalho durante a pandemia e do uso adequado de equipamentos de proteção individual.
Ximenes Neto FRG et al.^44^, 2020, Brasil	Descrever as ações estratégicas de coordenação do cuidado, monitoramento e vigilância dos casos da covid-19 na atenção primária à saúde.	Relato de experiência	Destacou-se a importância do isolamento social horizontal e o isolamento domiciliar dos casos positivos; uso de tecnologias digitais no território para divulgação das ações sobre a prevenção e a efetivação do teleatendimento; fortalecimento das ações intersetoriais entre saúde, educação e assistência social, e a estruturação da Rede de Atenção à Saúde.
Coma E et al.^46^, 2020, Espanha	Analisar o impacto da epidemia de covid-19 e das medidas de lockdown nos indicadores de qualidade da atenção à saúde e no controle de doenças crônicas.	Descritivo retrospectivo	Foram avaliados 34 indicadores de padrão de qualidade e 85% deles apresentaram resultado negativo no mês de março de 2020 e 68% em abril de 2020, quando comparados ao mesmo período de 2019. Destacam-se os indicadores de tratamento dos quais 100% apresentaram efeitos negativos; os de acompanhamento com 80% de efeitos negativos e os indicadores de controle das doenças crônicas, 90% apresentaram efeito negativo.
Garg S et al.^47^, 2020, Índia	Determinar a preparação das unidades de saúde da atenção primária para a prestação de serviços ambulatoriais seguros durante a pandemia de covid-19 na Índia.	Estudo transversal	Foram avaliados 51 serviços de atenção primária. Identificação de déficits na infraestrutura e controle de infecção. Redução dos atendimentos às doenças crônicas não transmissíveis, imunização, pré-natal e saúde materno-infantil, foram os mais afetados. Em contrapartida, nos locais de triagem para sintomas de gripe os atendimentos aumentaram.
Danhieux K et al.^48^, 2020, Bélgica	Examinar como a prestação de cuidados de atenção primária às condições crônicas foram afetadas durante a pandemia na Bélgica.	Estudo qualitativo	Houve mudanças na organização da atenção à saúde, com enfoque nos casos suspeitos de covid-19 e queda nos atendimentos às condições crônicas, uso de teleatendimento; a maioria dos profissionais entrevistados não realizou estratificação de risco e busca ativa dos pacientes de maior risco; uso de teleatendimento para avaliação e prescrição de medicamentos e não para o monitoramento das condições crônicas. Houve grave interrupção na prestação de cuidados crônicos.

APS: atenção primária à saúde.

Os estudos avaliados nesta revisão trouxeram um panorama mundial de como os profissionais e os serviços da atenção primária à saúde se organizaram para o enfrentamento da pandemia de covid-19, com destaque para o uso de tecnologias e da telemedicina. A alternativa de modalidade de atendimento e monitoramento dos casos por meio de aplicativos, telefone e plataformas online também foram amplamente abordados nos estudos, aparecendo em 17 (71%) estudos (Quadro 2).

As readequações adotadas na infraestrutura e fluxos de atendimentos, a exemplo da adoção de unidades específicas no atendimento para covid-19, separação de fluxos infectado/não infectado nas unidades de saúde, readequação do processo de trabalho e infraestrutura foram descritos em 13 (54%) estudos (Quadro 2).

A importância da educação em saúde sobre a covid-19 com a comunidade e com a própria equipe foi abordada em três estudos (12,5%); outras três pesquisas (12,5%) analisaram os cuidados direcionados aos pacientes crônicos durante a pandemia. Dois estudos (8,5%) investigaram a organização dos serviços e profissionais da APS, com base em experiências anteriores como na pandemia de influenza H1N1 (Quadro 2).

## DISCUSSÃO

Nos 24 artigos selecionados neste estudo destaca-se a abordagem sobre a reorganização dos serviços e dos profissionais de saúde da APS, além da vigilância no monitoramento dos casos de covid-19 para o atendimento de casos suspeitos ou confirmados da doença.

Ressalta-se que, no Brasil, em março de 2020, após a declaração da transmissão comunitária da doença causada pelo novo coronavírus em todo o território nacional, a Secretaria de Vigilância em Saúde do Ministério da Saúde realizou a adaptação do Sistema de Vigilância de Síndromes Respiratórias Agudas, visando orientar o Sistema Nacional de Vigilância em Saúde para a circulação simultânea do SARS-CoV-2, influenza e outros vírus respiratórios, no âmbito da Emergência de Saúde Pública de Importância Nacional^12^.

Diante de um cenário de enfrentamento de uma doença que foi considerada uma emergência de saúde pública, destaca-se o importante papel da vigilância em saúde na notificação, investigação e monitoramento dos casos supeitos e confirmados por covid-19^13^. As ações de vigilância em saúde no território mostra-se como um ponto essencial da organização da APS nesse novo cenário, por meio do desenvolvimento de ações que possibilitem a identificação precoce dos casos suspeitos, notificação imediata, busca ativa de contatos, reforço ao isolamento domiciliar dos casos e de seus contatos, educação em saúde e apoio aos grupos vulneráveis do território^14^.

É sabido que a pandemia da covid-19 resultou em um aumento da demanda assistencial sobre o SUS, fazendo o Ministério da Saúde reforçar o estabelecimento da organização da rede de atenção e dos fluxos, tanto para pessoas com síndrome gripal, incluindo a covid-19, quanto para as pessoas que necessitassem de acompanhamento por outras condições e agravos de saúde^12^.

Dessa forma, a atuação da APS, no contexto da pandemia de covid-19 no Brasil, evidenciou que a principal estratégia de enfrentamento foi a reorganização do processo de trabalho dos profissionais de saúde, destacando-se as atividades de educação em saúde, por construir saberes que propiciam o autocuidado^15^. Todos os países tiveram que se reorganizar e fortalecer a capacidade de resposta dos serviços da atenção primária à saúde, de acordo com suas políticas públicas, além de garantir a assistência às outras necessidades de saúde da população^16^.

Além disso, no Brasil, constantes atualizações de guias de vigilância epidemiológica foram realizadas para nortear o combate à covid-19^12^. Nesse contexto, o Ministério da Saúde publicou o “Protocolo de Manejo Clínico da covid-19 na Atenção Primária à Saúde” recomendando a reorganização dos serviços e do processo de trabalho da APS para o enfrentamento da pandemia. Esse protocolo definiu medidas para a prevenção de contágio nos serviços de saúde, modelos para estratificação da gravidade de casos suspeitos, ações para acompanhamento terapêutico e isolamento domiciliar dos casos leves, além da definição de medidas para estabilização e encaminhamento aos serviços de maior complexidade e ações de promoção de medidas de prevenção comunitária^17^.

A reorganização da estrutura física dos serviços da APS foi uma das principais estratégias adotadas a fim de minimizar os riscos de contágio dentro das unidades de saúde, além de propiciar agilidade aos atendimentos, evitar o contato entre os usuários suspeitos e não suspeitos de covid-19, bem como a proteção dos profissionais, maximizando a eficiência dos serviços prestados.

Estudos realizados no Brasil, EUA, Bélgica e Islândia adaptaram as unidades de saúde para manter o distanciamento físico, adotaram a identificação imediata de casos sintomáticos durante o acolhimento na recepção, limitação do número de acompanhantes, consultórios separados para usuários sintomáticos e assintomáticos e reorganização dos atendimentos dos grupos prioritários assistidos pelo serviço^18^.

Em Diadema (SP) a gestão da saúde optou por descentralizar os atendimentos aos sintomáticos respiratórios em todas as unidades da APS, apoiando-se na característica da capilaridade da atenção primária com as equipes de saúde da família , com isso, todos os serviços desse nível de assistência acolheram essa demanda^24^.

Em Florianópolis (SC), Sobral (CE) e Belo Horizonte (MG), as unidades de saúde que possuíam espaço físico adequado, também organizaram os fluxos de atendimentos separadamente, ou seja, usuários suspeitos e confirmados eram acolhidos e atendidos em locais separados das demais demandas^25^.

Por outro lado, foi verificado a experiência de serviços de saúde que optaram por centralizar os atendimentos de sintomáticos respiratórios em uma ou mais unidades de saúde específica. A organização desses serviços apresentou algumas similaridades, como o respeito às instruções aos usuários sobre os cuidados de higienização pessoal e uso de máscara, além de possuírem espaços físicos redesenhados para respeitar o distanciamento físico^28^.

Na Inglaterra, foram criadas unidades de atendimento específico para pessoas com diagnóstico de covid-19, além do monitoramento domiciliar de usuários que não tinham condições de se deslocarem para um serviço de saúde ou não necessitavam de atendimento hospitalar^30^.

Já em Rochester (Minnesota-EUA), essa mesma estratégia foi adotada para evitar o contato de casos confirmados ou em investigação com os outros usuários que necessitavam de atendimento. Das cinco unidades de saúde da cidade, uma delas foi readequada para atender exclusivamente usuários com diagnóstico de covid-19 ou com sintomas de problemas respiratórios, e inicialmente foi adotada a estratégia de triagem por telefone, identificando se a pessoa necessitava de atendimento hospitalar ou poderia ser atendido na unidade covid-19^28^.

Diante da necessidade de readequação do atendimento presencial nas unidades de saúde no contexto pandêmico, as estratégias para a assistência à distância surgiram como alternativa, tanto para realizar o monitoramento de usuários com o diagnóstico da doença quanto para o acompanhamento das outras demandas^6,14,24,31^. Assim, foram inseridas na rotina dos profissionais de saúde o teleatendimento e monitoramento remoto, visando a redução do número de usuários dentro dos serviços e encaminhamento para a unidade de saúde apenas quando necessário; tais medidas foram adotadas com o objetivo de reduzir a circulação do SARS-CoV-2^20^.

Na Austrália, a atenção primária reorganizou a assistência por meio da adoção de teleconsultas, call centers para triagem de pessoas com sintomas respiratórios e orientações, além do estabelecimento de uma rede nacional de unidades respiratórias em complemento. Também foram realizados treinamentos online para os profissionais da saúde e divulgação das medidas de proteção à saúde das comunidades remotas de aborígenes e da população das ilhas do Estreito de Torres^42^.

No Reino Unido, o número de consultas à distância dobrou, ao passo que as consultas presenciais e visitas domiciliares diminuíram, resultando no atendimento de três quartos dos usuários recepcionados pela atenção primária à distância^30,38^. Já na Islândia, houve um aumento de 127% nas consultas realizadas por telefone ou online, verificando ainda elevação no número de prescrições de medicamentos e consultas à distância^18^. Esse padrão de crescimento também foi observado na França, na Itália e na Bélgica, uma vez que a grande maioria dos médicos generalistas adaptaram suas atividades para a modalidade remota^19,29,40^.

Na Itália, o monitoramento e acompanhamento de usuários com covid-19 foi domiciliar, de forma remota, por meio de aplicativos de mídia social, sendo que esse acompanhamento era realizado duas vezes ao dia, monitorando inclusive os sinais vitais dos pacientes, mensurados por meio de smartphones. Em situações em que fosse identificada dificuldade de o usuário realizar o acompanhamento dos sinais vitais por meio desses dispositivos tecnológicos, era emprestado oxímetro de pulso, para que pudesse monitorar a saturação e a frequência cardíaca no domicílio^40^.

Embora o teleatendimento possibilite a continuidade da assistência à distância, essa mudança pode dificultar a prática dos médicos generalistas, uma vez que ocorre a perda de comunicação não verbal, além da capacidade limitada de alguns usuários em articular suas necessidades e a comunicação intercultural associada aos problemas de linguagem, que surgem como barreiras no atendimento^34^.

Estudo realizado com a população idosa atendida pela APS no Reino Unido mostrou que é pertinente que os profissionais de saúde tenham ciência de que consultas remotas, principalmente se tratando de videochamadas, podem representar uma barreira adicional ao acesso, quanto aos cuidados para grupos de classes sociais desfavorecidas, que possuem acesso limitado à internet, smartphones e demais tecnologias^38^.

A pandemia propiciou inovação no atendimento realizado pela APS, de forma a se adequar às medidas de distanciamento físico. Entretanto, a atenção primária deve avaliar a eficiência do atendimento remoto e reconhecer as possíveis dificuldades de uma população no que tange às tecnologias necessárias.

No âmbito da APS, as ações de educação se fortaleceram e se destacaram no cuidado oferecido pelos profissionais da saúde direcionado para limitar a propagação da covid-19^22,23,32,34,43,44^. Nesse sentido, destaca-se a dificuldade de se lidar com rumores da comunidade e informações enganosas, implicando diretamente no processo de cuidado da população durante a pandemia, situação que evidencia a importância da integração do suporte de tecnologia e divulgação de orientações confiáveis^32^.

A adesão às medidas de prevenção contra a covid-19 está relacionada às ações de educação em saúde realizadas pelos profissionais de saúde. Nesse sentido, enfatiza-se a importância de tecnologias digitais para disseminação de informações pelas redes sociais sobre a prevenção da doença, além de reforçarem o papel fundamental dos agentes comunitários de saúde na educação em saúde de uma comunidade, principalmente no combate às *fake news* e na mediação da APS com a comunidade^22,43^.

Em um município brasileiro, as equipes de saúde da família, juntamente com os agentes comunitários, realizaram atividades de educação em saúde direcionadas à população. Essas ações tinham como objetivo orientar a população sobre às medidas preventivas contra a covid-19, além de divulgar os dados epidemiológicos por meio da distribuição de panfletos, carro de som, rádio, redes sociais, contato telefônico. Entretanto, o número de casos suspeitos de covid-19 não foi reduzido, evidenciando a importância da adesão das pessoas às medidas de prevenção^19^. Para que as ações de educação em saúde atinjam seus objetivos é necessário o desenvolvimento de estratégias diversas, como forma de atravessar barreiras sociais e culturais que determinam as escolhas dos indivíduos^22,45^.

Durante a pandemia de covid-19, também se destaca o adiamento e a redução no acompanhamento de usuários com diagnóstico de doença crônica não transmissível (DCNT), de maneira global, uma vez que parte desses cuidados não são urgentes^21,22,29,32,34,46^. Em Mascate (Omã), todas as unidades de saúde suspenderam a assistência presencial de usuários com DCNT no início da pandemia^32^. Já na Espanha e na Bélgica, embora não tenha ocorrido a suspensão, houve uma significante diminuição do acompanhamento de usuários com diabetes mellitus e hipertensão arterial sistêmica^46,48^.

Quanto à população infantil, foi observado uma redução na administração de vacinas em crianças na Espanha^46^ e uma interrupção na operação de serviços ambulatoriais relacionados à saúde materno-infantil na Índia^47^. Na França, o atendimento ambulatorial de outras demandas da população foi mantido, enquanto os atendimentos de covid-19 foram direcionados e concentrados em centros específicos para a assistência a esse público^16^.

O adiamento da assistência a usuários com DCNT, puericultura e redução da cobertura vacinal podem acarretar consequências que se estenderão após a crise pandêmica da covid-19, gerando uma sobrecarga nos sistemas de saúde^21,29,34,46^. Por outro lado, a redução do comparecimento de usuários com DCNT às unidades de saúde pode estar relacionada às recomendações das autoridades de saúde para que as pessoas permanecessem em casa e procurassem esses serviços somente se apresentassem sintomas de covid-19^29^.

Embora os profissionais de saúde que atuam na atenção primária tenham se organizado de maneira rápida em resposta ao início da pandemia, o direcionamento da assistência à covid-19 pode causar complicações à saúde de uma parcela da população que teve seus cuidados adiados ou suspensos, além de onerar o sistema de saúde.

Diante dessa situação, a garantia do cuidado integral, durante a pandemia, se tornou um grande desafio para a APS, como consequência da valorização do cuidado nos serviços hospitalares e detrimento das outras demandas da população^5,22,43,45^. Portanto, a pandemia reafirma a necessidade do fortalecimento do protagonismo da atenção primária na organização dos serviços de saúde no SUS, como forma de garantir a otimização de gastos, redução de hospitalizações, tanto pela covid-19 quanto por outras causas sensíveis a esse nível de assistência.

No Brasil, o Ministerio da Saúde publicou manuais e portarias que orientaram como os serviços de saúde e profissionais da APS deveriam proceder, além disso, as secretarias estaduais de saúde, juntamente com o Conselho Nacional de Secretarios Estaduais de Saúde , apoiaram os gestores municipais na tomada de decisão a respeito dessa reestruturação dos serviços; porém, apesar das normativas, cada município se adequaou conforme sua realidade local, influenciado tanto por questões epidemiológicas, quanto políticas e financeiras.

O fato de a atenção primária desempenhar um papel ordenador na rede de saúde fortalece a sua importância no enfrentamento da pandemia, identificando precocemente os casos suspeitos, além de acompanhar os casos leves, identificar e encaminhar os casos graves, e contribuir na redução da sobrecarga dos serviços especializados e hospitalares, o que, consequentemente, leva à redução nos gastos públicos.

O presente estudo mostra um panorama de como os profissionais e serviços da atenção primária à saúde se organizaram para o enfrentamento da pandemia, abordando as readequações adotadas na infraestrutura e fluxos de atendimentos, como por exemplo a adoção de unidades específicas no atendimento para covid-19, separação de fluxos contaminado e não contaminado, a telemedicina como alternativa de modalidade de atendimento e monitoramento dos casos por meio de aplicativos e via telefone. Entretanto, persiste lacunas na literatura como a avaliação do impacto dessas ações e a sua efetividade na mitigação da transmissão da covid-19; análise das consequências para os usuários que tiveram seus atendimentos adiados ou reduzidos, a exemplo das DCNT, acompanhamento de puericultura, pré-natal e cobertura vacinal durante o período pandêmico.
